# Teaching of evidence-based medicine to medical students in Mexico: a randomized controlled trial

**DOI:** 10.1186/1472-6920-12-107

**Published:** 2012-11-06

**Authors:** Melchor Sánchez-Mendiola, Luis F Kieffer-Escobar, Salvador Marín-Beltrán, Steven M Downing, Alan Schwartz

**Affiliations:** 1 Department of Medical Education, UNAM Faculty of Medicine, Mexico City, Mexico; 2 Department of Biomedical Informatics, UNAM Faculty of Medicine, Mexico City, Mexico; 3 Department of Pediatrics, Central Military Hospital, Mexico city, Mexico; 4 Department of Medical Education, College of Medicine, University of Illinois at Chicago, Chicago, IL, USA

**Keywords:** Evidence-based medicine, Undergraduate medical education, Curriculum development, Educational assessment, Critical appraisal skills

## Abstract

**Background:**

Evidence-Based Medicine (EBM) is an important competency for the healthcare professional. Experimental evidence of EBM educational interventions from rigorous research studies is limited. The main objective of this study was to assess EBM learning (knowledge, attitudes and self-reported skills) in undergraduate medical students with a randomized controlled trial.

**Methods:**

The educational intervention was a one-semester EBM course in the 5^th^ year of a public medical school in Mexico. The study design was an experimental parallel group randomized controlled trial for the main outcome measures in the 5^th^ year class (M5 EBM vs. M5 non-EBM groups), and quasi-experimental with static-groups comparisons for the 4^th^ year (M4, not yet exposed) and 6^th^ year (M6, exposed 6 months to a year earlier) groups. EBM attitudes, knowledge and self-reported skills were measured using Taylor’s questionnaire and a summative exam which comprised of a 100-item multiple-choice question (MCQ) test.

**Results:**

289 Medical students were assessed: M5 EBM=48, M5 non-EBM=47, M4=87, and M6=107. There was a higher reported use of the Cochrane Library and secondary journals in the intervention group (M5 vs. M5 non-EBM). Critical appraisal skills and attitude scores were higher in the intervention group (M5) and in the group of students exposed to EBM instruction during the previous year (M6). The knowledge level was higher after the intervention in the M5 EBM group compared to the M5 non-EBM group (p<0.001, Cohen's *d*=0.88 with Taylor's instrument and 3.54 with the 100-item MCQ test). M6 Students that received the intervention in the previous year had a knowledge score higher than the M4 and M5 non-EBM groups, but lower than the M5 EBM group.

**Conclusions:**

Formal medical student training in EBM produced higher scores in attitudes, knowledge and self-reported critical appraisal skills compared with a randomized control group. Data from the concurrent groups add validity evidence to the study, but rigorous follow-up needs to be done to document retention of EBM abilities.

## Background

Evidence-based medicine (EBM) has been defined as “the integration of the best research evidence with our clinical expertise and our patient’s unique values and circumstances”, and it has emerged as a core competency necessary for all healthcare professionals [1-3]. Its fundamental principles are: translation of uncertainty to an answerable clinical question, systematic retrieval of the best evidence available, critical appraisal for validity, relevance and applicability, use of results in practice and evaluation of its performance by the healthcare provider [4].

Several organizations, including the Institute of Medicine in the United States and the World Federation for Medical Education, have advocated the implementation of EBM educational interventions in medical under and postgraduate training [2,5].

The concepts related to EBM and its educational implications have disseminated rapidly in the last decade, and this change needs to be accompanied with strong educational research to document its effectiveness. The challenges of teaching EBM and the paucity of rigorous educational research publications have prompted some medical educators to question the evidence of EBM teaching effectiveness [6]. Nonetheless, the foundations of EBM that support clinical decision making are intuitively attractive to many clinicians and educators, since it integrates the educational process with clinical practice [4].

The quality of the evidence about EBM education is heterogeneous, as has been described in several editorials, narrative and systematic reviews [7-11]. The majority of reviews have included mostly studies in postgraduate health professionals, and some have included studies in both post and undergraduate students. Green reviewed 18 reports, mostly resident-directed small-group seminars with the objective of improving critical appraisal skills [12]. The most commonly used outcome measure was a multiple-choice exam, and 72% used a traditional journal club format as teaching strategy. Only seven of the 18 studies included in Green’s review analyzed the effectiveness of the intervention, five of these had some type of control group and only one was a randomized study. Just two studies used an outcome measure that had validity evidence, and measurement of change in behavior used only self-report in all five papers. The impact of the intervention was focused mainly on critical appraisal, and ranged from no effect to 23% absolute increase in scores [12].

The Cochrane Collaboration systematic review on the subject of teaching critical appraisal skills in health care, which excluded medical students, found three studies that met stringent pre-specified methodological criteria. These articles reported statistically significant improvements in participants' knowledge in domains of critical appraisal in two of the three studies [9]. Another systematic review by Coomarasamy focused on postgraduate clinicians, and found significant effects of EBM educational interventions in knowledge, and more limited in attitudes, skills and behavior [10,11].

Despite the increasing number of medical school and postgraduate programs that have introduced EBM in their curricula, most of the information about it has been reported as observational data and descriptive studies in the medical literature, or as unpublished observations that are disseminated in medical meetings or informal venues. There are few randomized controlled educational trials about EBM training effectiveness, and the majority have been done in residents or practicing physicians [9-14].

Undergraduate medical students can be a receptive population to EBM concepts, and they will be the practicing clinicians and clinical teachers in the future. There are several published studies that describe medical schools’ experiences introducing EBM in their curriculum and teaching these concepts to undergraduates, with variable outcomes [15-19]. This curricular change has not occurred in many of their developing country counterparts, with few published reports of the implementation of EBM curricula in these settings [20-23]. There is a need to implement EBM educational interventions in developing countries medical schools’ curricula, and to assess their impact with appropriate educational research designs.

The purpose of this study was to assess the educational effectiveness (attitudes, knowledge and skills) of an EBM course in undergraduate medical students.

## Methods

### Setting

The Mexican Army medical school trains career physicians for the national military healthcare system, and is located in Mexico City. It has a six year program, with a traditional curriculum: two years of basic sciences, three years of clinical sciences, and the sixth year is an internship period in the hospital. The school is a public institution funded by the federal government. Each yearly class is composed of about one hundred students, mostly middle- or low-socioeconomic class Hispanics.

### Overall study design and participants

#### Main outcomes and subjects

**Figure 1:**
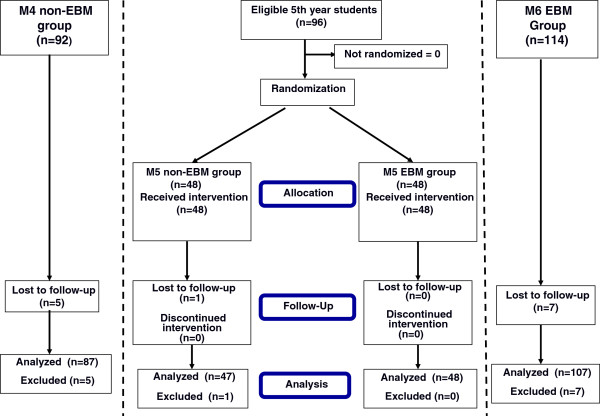
**Flow diagram of study participants.** Flow diagram summarizing the groups of medical students and the progress of their participation in the study. M4 non-EBM=4^th^ year students with no evidence-based medicine training; M5 EBM and M5 non-EBM=5^th^ year medical students with and without the evidence-based medicine course; M6 EBM=6^th^ year students exposed to the evidence-based medicine course during the year prior to assessment.

The core portion of the study was a randomized post-test only control group design, for the main outcomes: attitudes, knowledge and skills in EBM. Fifth year medical students were randomized in two groups, one of which was subjected to the educational intervention during the first semester of the academic year (M5 EBM), while the other half (M5 non-EBM) had an Aviation Medicine course (Figure 1). The rest of the 5^th^ year curriculum was similar in that semester. In the second semester the control group had the EBM course and the intervention group had the Aviation Medicine course. The randomization was done by the medical school with a computer generated list, using the block randomization method with blocks of two to ensure equal sample sizes [24].

#### Simultaneous validation

Quasi-experimental static-groups comparisons were added besides the randomized trial, with a more junior group of 4^th^ year students not yet exposed to the EBM intervention (M4 non-EBM) and a more senior group in 6^th^ year that had the EBM course during the previous year (M6 EBM). The 4^th^ year students had courses on Medical Informatics, Statistics, Research Methodology and Epidemiology, which are taught by information technology professionals, statisticians, epidemiologists and basic-science researchers, most of them with no clinical background. The 6^th^ year students were in the hospital internship and all of them had the EBM course during the previous year (half of them six months and half one year before the evaluation). These comparison groups were included to acquire more information from our population in concurrent groups and increase the validity of the study, addressing the history, maturation and contamination threats to validity and exploring the potential EBM knowledge in more senior students [25-27] (Figure 1).

The outcomes were measured in all groups at the end of the first semester of the academic year, after the EBM course ended. All the fifth, fourth and sixth year students were asked to participate in the study, about one hundred students per class.

### Intervention

The educational intervention was a one semester EBM course formally included in the medical school curriculum, with 14 two-hour weekly sessions. The course faculty were six professors trained in EBM teaching, all board-certified physicians with clinical practice, one of them with a postgraduate degree in health professions education and faculty development training in EBM education at McMaster University Faculty of Health Sciences in Canada. The course faculty had more than six years of experience teaching EBM to undergraduate medical students, residents of several specialties, and providing faculty development EBM workshops to teachers of several medical specialties. The EBM course teachers were not involved in the training of the 4^th^ year students, but they participated in the training of the 6^th^ year interns. The EBM program was linked with the internship program and the residency programs in the hospital, through the medical school curricular committee and the University Postgraduate Studies Division.

The course instructional strategies included large-group interactive sessions, small-group problem-solving activities, individual and group assignments, and informatics laboratory sessions. Traditional EBM resources were used as course bibliography, including Straus’ book [1] and an EBM text in Spanish written by the course professors [28]. The content and learning objectives of the course are outlined below.

1. Clinical decision making in medicine

 • List and define the main difficulties for objective decision making in medicine as defined by Eddy

 • Describe the components of a decision in medicine as defined by Eddy

 • Apply the concepts of anatomy of a decision as defined by Eddy in the analysis of a clinical problem

2. Uncertainty and probability in medicine

 • Define the concepts of uncertainty, probability and odds

 • Understand the relevance of uncertainty in clinical practice

 • Understand the limitations of personal experience in the estimation of probability, as related to diagnosis

 • Define the heuristics used in medicine (representativeness, availability, anchor and adjustment) and list the cognitive errors a clinician can make when misapplying them

 • Apply the concepts of heuristics in new clinical problems, and discuss the effects of their inappropriate use

3. Bayes’ theorem

 • Define Bayes’ theorem

 • Define pre-test and post-test probability

 • Define the concepts of diagnostic and therapeutic threshold

 • Explain the utility of Bayes' theorem in clinical medicine, mainly in diagnosis

 • List the limitations of Bayes' theorem in clinical practice

 • Apply Fagan’s nomogram to use Bayes' theorem in a diagnostic problem

 • Apply the concepts of diagnostic and therapeutic threshold to a clinical problem

4. Principles of Evidence Based Medicine

 • Describe the history and origin of EBM

 • Define the concept of EBM

 • List the five steps of EBM, and apply them in a clinical problem

 • Explain the importance of EBM in clinical practice

5. Reflective medical practice

 • Define the concept of reflection and reflective practitioner

 • Define reflection-in-action and reflection-on-action

 • Apply these concepts in a clinical scenario

6. Clinicians’ information needs

 • Understand the magnitude of physician information needs

 • Understand the literature that describe how clinicians underestimate their information needs

 • Define the percentage of occasions when clinicians recognize and act upon perceived information needs

7. Clinical questions

 • Define the concepts of background and foreground questions

 • Understand the advantages of structuring questions generated during clinical work

 • List the four components of a foreground clinical question (PICO)

 • Apply these concepts in developing questions from clinical problems

 • List the types of clinical questions (diagnosis, therapy, prognosis, harm, etiology)

8. Sources of biomedical information

 • List the different sources of biomedical information available

 • Identify the advantages and disadvantages of each source (textbooks, paper and electronic journals, original research papers)

 • Understand the origin, development, cost, and availability of sources of information

9. The Cochrane Collaboration

 • Describe the history and origin of the Cochrane Collaboration (CC)

 • List the components of the Cochrane Library, and the sources where it’s available

 • Understand the mission, logistics and work of the CC

 • Perform effective searches for systematic reviews on the Cochrane Library

 • Understand the advantages and limitations of the CC

 • Use the Cochrane Library to solve a clinical problem

10. Search strategies to find the best medical scientific evidence

 • List the main medical databases, and identify their relevance and location

 • Describe the history of Medline

 • Define MeSH terms, Boolean operators, search engine

 • Design search strategies to find valid evidence

 • Use PubMed Clinical Queries

 • Perform effective searches of scientifically valid papers using PubMed, Cochrane Library, OVID Core Medical Library

 • Understand the advantages and disadvantages of searching the different electronic medical databases and the Internet general purpose searching engines

11. Critical appraisal of the medical literature: Users’ Guides to the Medical Literature

 • Describe the origin and history of the Users’ Guides series to appraise the medical literature

 • List and understand the different hierarchies of evidence, study designs, grades of evidence

 • Understand the relevance of using the original medical literature to solve clinical problems

 • List and understand the three different steps to appraise a research article: internal validity, magnitude of the results and external validity

12. How to appraise an article about therapy

 • Describe the criteria for internal validity of a therapy article

 • Define randomized controlled trial, bias and random error, allocation concealment, double-blind, intention-to-treat analysis, odds ratio, relative risk, relative risk reduction, absolute risk reduction, number needed to treat, confidence intervals, p values, power and sample size, type I and II errors

 • Understand the importance of all the previously defined concepts to apply in a therapy article

 • Calculate OR, RR, RRR, ARR and NNT from a published therapy article

 • Use a therapy article to solve a clinical problem

 • Understand the concepts of external validity of a research paper, related to therapy

13. How to appraise an article about a diagnostic test, the Rational Clinical Examination Series

 • Describe the criteria for internal validity of a diagnostic test article

 • Define pre-test and post-test probability, sensitivity, specificity, likelihood ratios, positive and negative predictive value, accuracy

 • Understand the importance of all the previously defined concepts to apply a diagnosis article

 • Calculate sensitivity, specificity, likelihood ratios from a published diagnosis article

 • Use a diagnosis article to solve a clinical problem

 • Understand the concepts of external validity of a research paper, related to diagnosis

 • Describe the origin and evolution of the Rational Clinical Examination JAMA series

 • Use a JAMA Rational Clinical Examination paper to solve a clinical problem

14. How to appraise a Systematic Review or Meta-analysis

 • Define meta-analysis, systematic review (qualitative and quantitative)

 • Describe the advantages and limitations of systematic reviews and meta-analysis

 • Describe the criteria for internal validity of a systematic review article

 • Define bias and random error, odds ratio, relative risk, relative risk reduction, absolute risk reduction, number needed to treat, confidence intervals, forest plot, effect size

 • Understand the importance of all the previously defined concepts applied to a systematic review article

 • Calculate OR, RR, RRR, ARR and NNT from a published systematic review article

 • Use a systematic review article to solve a clinical problem

 • Understand the concepts of external validity of a systematic review

15. Clinical practice guidelines

 • Define clinical practice guidelines

 • Describe the sequence of developing an evidence-based clinical practice guideline

 • Understand the advantages and limitations of a clinical guideline

 • Describe and understand the internal validity requirements of a clinical guideline article

 • List the available resources for clinical guidelines

 • Use a clinical practice guideline to solve a clinical problem

### Outcomes and Instrumentation

The assessed outcomes were attitudes, knowledge and skills related to EBM. Two instruments were used: Taylor’s questionnaire, a published instrument designed to evaluate the effectiveness of evidence-based medicine teaching [29] and a 100 multiple-choice question test developed specifically for this study.

Taylor’s instrument was categorized as a level 1 instrument in a systematic review of tools to evaluate EBM education, since it has reasonable psychometric properties, has been evaluated for validity from at least three sources of evidence, and is recommended for use in the summative evaluation of individual trainees [30]. The instrument includes items to assess critical appraisal skills, use of evidence behaviors, knowledge and attitudes regarding evidence-based clinical practice [29]. The attitude portion of the questionnaire includes statements related to the use of scientific evidence using a Likert scale. Each statement is scored on a five point scale, responses are added to obtain a total attitude score, and the range of scores is 7 to 35. To determine an overall score for the confidence in critical appraisal skills section, six statements were scored using a scale where “Very confident” was assigned a score of 5, “Not at all confident” a score of 1, and “Don’t know” a score of 0. The scores of the six questions were added, providing a global critical appraisal skills confidence score, where 5 indicated “little or no confidence” and 30 indicated “complete confidence”.

The knowledge part of the questionnaire includes six multiple true-false questions, each with three items, using ‘true’, ‘false’ or ‘don’t know’ response categories. Correct responses to the knowledge questions have a score of 1, incorrect responses are negatively scored (−1) to try to prevent guessing, and the ‘don’t know’ response has a score of 0. The knowledge scores were added in an overall knowledge score, with a possible range of −18 to +18. In a previous paper, we translated the questionnaire to Spanish with the author’s permission, and verified it with backtranslation [21].

The other instrument used was the final summative test of the Evidence-Based Medicine Course. This instrument was developed, administered, scored, and analyzed following the 12 steps for effective test development described by Downing [31]. Item analysis was performed on a pilot application of the test with ITEMAN for Windows (Assessment Systems Corporation, St. Paul, MN), and the information obtained was used to improve the instrument for this study, choosing the better-performing items and preserving content validity. The pilot application of the original 140-items EBM test was done in 57 examinees, and had a Cronbach’s alpha of 0.82. Using the item analysis information 100 multiple-choice questions (MCQ) were selected by the test developers for the final version of the instrument.

The instruments were applied to the students on three consecutive weeks. The students had up to three hours to answer the test and the questionnaire, to minimize the risk of a speeded examination. Taylor’s questionnaires data sheets were captured in a Microsoft Excel spreadsheet. Op-scan answer sheets for item analysis were used for the EBM MCQ test.

### Statistical analysis

The piloting of the EBM MCQ test provided preliminary data for differences and standard deviation, and sample size calculation was performed for the primary hypothesis of knowledge increase with a power of 0.90 (beta error of 0.10), two-sided alpha error of 0.05. After a thorough review of the published studies that included magnitude of EBM knowledge differences in undergraduate medical students, and careful consideration by the course faculty of the smallest meaningful difference (SMD) in this parameter, it was estimated that a difference of 10 questions between the intervention group and the control group would be reasonable. Using this estimate, about 31 students per group would be necessary to detect an effect size of 0.5 or larger.

SPSS for Windows 15.0 and Instat 3.0 for the Macintosh were used for data analysis. The comparison of the use of evidence items in Taylor’s questionnaire between M5 and M5 non-EBM students was done with the non-parametric Mann–Whitney U test. The attitude and critical appraisal confidence scores measured with Taylor’s instrument were compared among groups using the Kruskal-Wallis with Dunn’s multiple comparison test. The groups’ knowledge test scores with both instruments were compared with one-way analysis of variance, with planned comparisons. A *p-*value of less than 0.05 was considered statistically significant. Cohen’s *d* with pooled standard deviations was calculated as a measure of effect size for the critical appraisal skills, attitude and knowledge scores among groups [32]. Item analysis of the EBM Test data was performed with ITEMAN for Windows 3.2, (Assessment Systems Corporation, St. Paul, MN http://www.assess.com).

### Ethical aspects

The instruments did not have individual student identifiers, to eliminate the risk of potential harm to the participants. This study was reviewed by the Institutional Review Board of the Office for the Protection of Research Subjects of the University of Illinois at Chicago, and the Research Committee of the Mexican Army Medical School, and was considered to be in the exempt category for individual written informed consent.

## Results

### Subjects

The flow diagram of the study participants throughout the trial is outlined in Figure 1. A total of 289 medical students were assessed. One student from the M5 non-EBM group was sick on the assessment day. Five subjects in the M4 non-EBM and 7 subjects in the M6 EBM groups didn't participate because they were on clinical duties on the testing day.

The students’ age (mean±SD) per group was: M4= 21.5±1.8, M5 EBM=22.8±2.0, M5 non-EBM=22.4±2.2 and M6=23.5±1.9 years. The groups’ gender composition was similar, with a predominance of women over men (about 60/40).

### Use of the evidence

**Figure 2:**
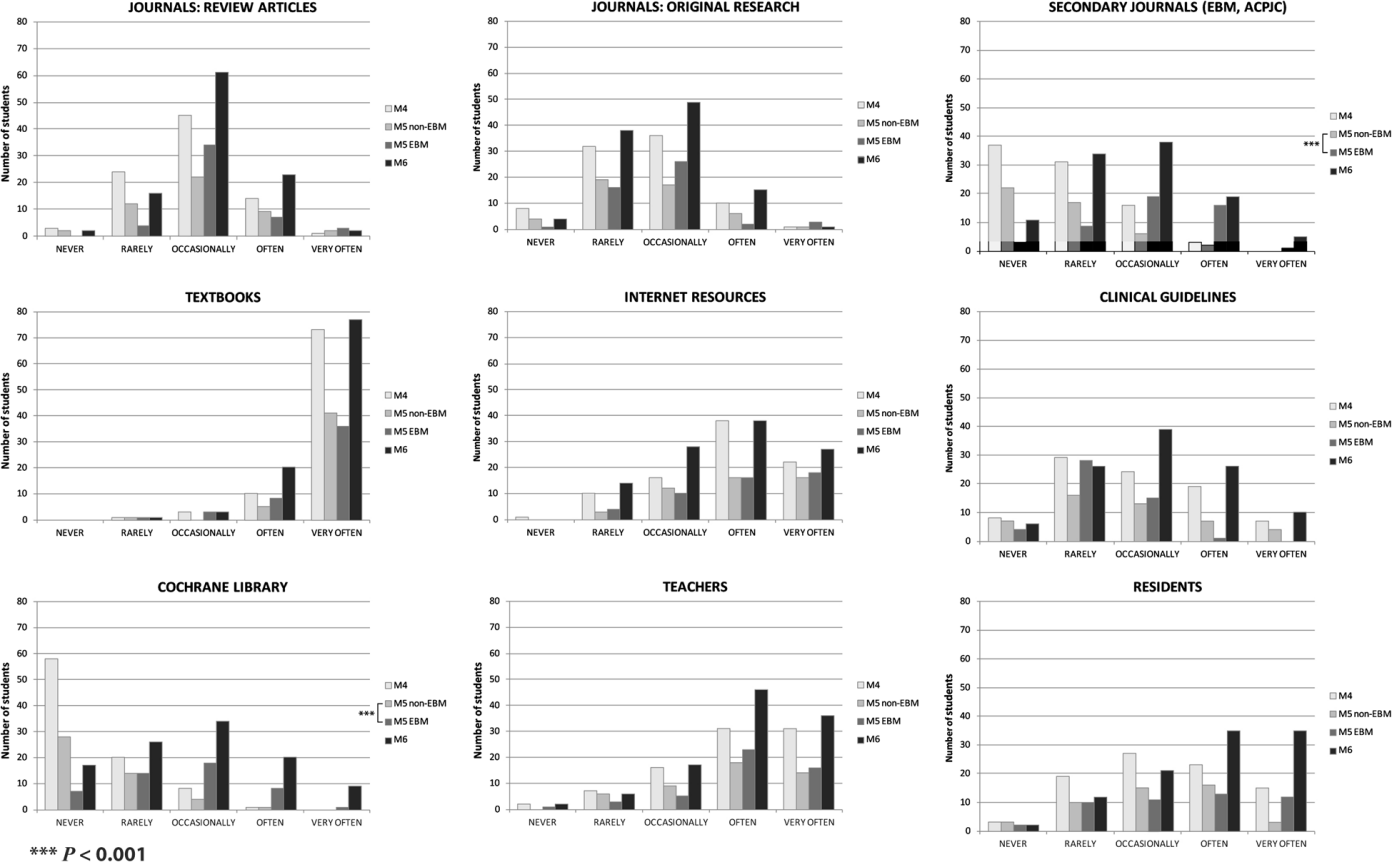
**Use of evidence to keep up to date.** Distribution of answers to the question: "what type of resources do you use to keep up to date?" in the different medical student groups. (M4=4^th^ year students with no evidence-based medicine training; M5 EBM and M5 non-EBM=5^th^ year medical students with and without the evidence-based medicine course; M6=6^th^ year students exposed to the evidence-based medicine course during the year prior to assessment; EBM=Evidence-Based Medicine; ACPJC=American College of Physicians Journal Club). *** = P<0.001 Mann–Whitney U test for the M5 vs. M5 non-EBM comparison.

**Figure 3:**
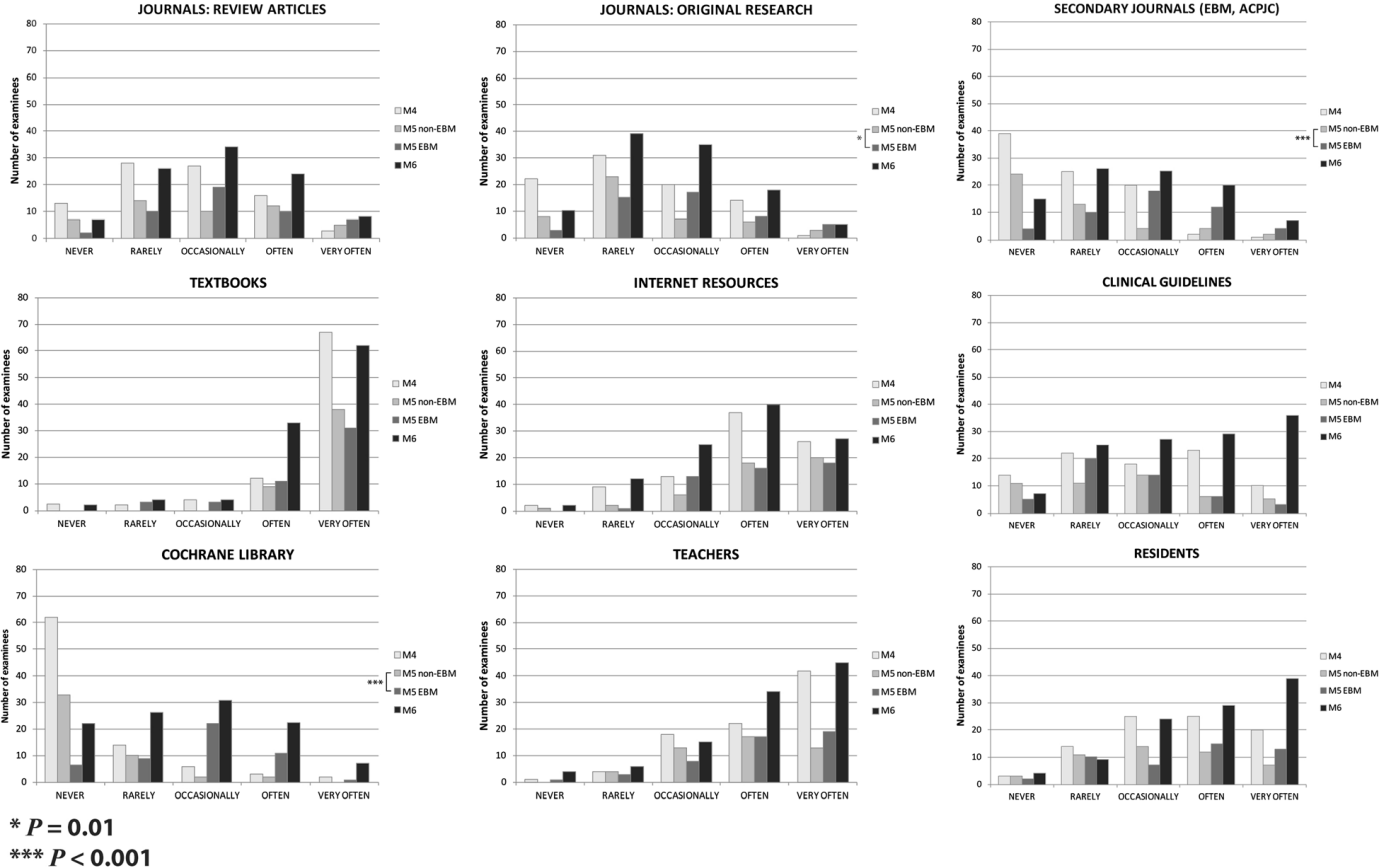
**Use of evidence to solve a health problem.** Distribution of answers to the question: "what type of resources do you use to solve a specific health problem?" in the different medical student groups. (M4=4^th^ year students with no evidence-based medicine training; M5 EBM and M5 non-EBM=5^th^ year medical students with and without the evidence-based medicine course; M6=6^th^ year students exposed to the evidence-based medicine course during the year prior to assessment; EBM=Evidence-Based Medicine; ACPJC=American College of Physicians Journal Club). * =*P*<0.01 Mann–Whitney U test for the M5 vs. M5 non-EBM comparison.*** = *P*<0.001 Mann–Whitney U test for the M5 vs. M5 non-EBM comparison.

The use of scientific evidence explored in the first section of Taylor’s questionnaire, includes two main questions: *“What type of resources do you use to keep up to date?”* and *“What type of resources do you use to solve a specific health care problem?”* The answers by group and type of resource are presented in Figures 2 and 3.

In the use of information resources to keep up to date and to solve a specific health care problem, the pattern of responses was the same. The answers were similar among the four student groups regarding the use of review articles, original research journals, textbooks, Internet resources and teachers, but there were statistically significant differences in the use of secondary journals (e.g. American Journal of Physicians Journal Club) and the Cochrane Library. The experimental group (M5 EBM) had a higher reported use of original research articles to solve a specific health problem than the randomized comparison group (M5 non-EBM) (*P*<0.01). The M5 EBM and M6 groups reported a higher use of secondary journals than the M4 and the M5 non-EBM groups, and a similar pattern of response was found in the use of the Cochrane Library (*P*<0.001) (Figures 2 and 3).

### Confidence in critical appraisal skills

**Figure 4:**
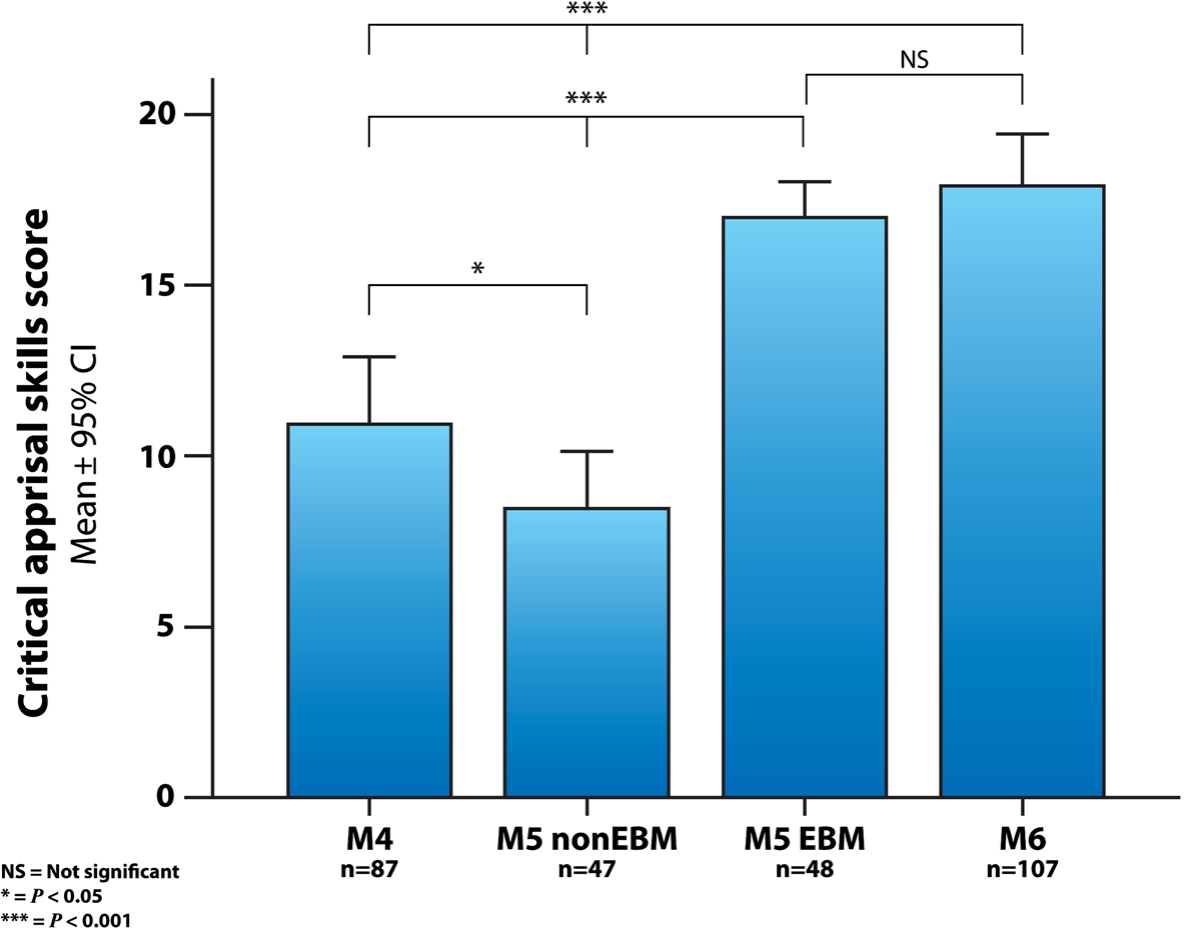
**Critical appraisal skills scores.** Critical appraisal confidence scores in the different groups of medical students, measured with Taylor’s questionnaire. (M4=4^th^ year students with no evidence-based medicine training; M5 EBM and M5 non-EBM=5^th^ year medical students with and without the evidence-based medicine course; M6=6^th^ year students exposed to the evidence-based medicine course during the year prior to assessment; CI=confidence interval).

There was a higher confidence level of critical appraisal skills in all items in this section of Taylor’s instrument (assessing study design, evaluating bias, evaluating statistical tests), in the intervention group (*P*<0.001). The critical appraisal confidence global scores for the different study groups were as follows: M4=11.7±6.3 (mean±SD), M5 non-EBM=8.4±5.7, M5 EBM=17.1±3.6 and M6= 16.8±4.9. The summary data for each group is shown in Figure 4, where the experimental group (M5 EBM) had higher scores than the randomized control group (M5 non-EBM) and the M4 comparison group (*P*<0.001). The M4 score was slightly higher than the M5 non-EBM group (*P*<0.05), and the M6 group had higher scores than M4 and M5 non-EBM (*P*<0.001).

### Attitudes

**Figure 5:**
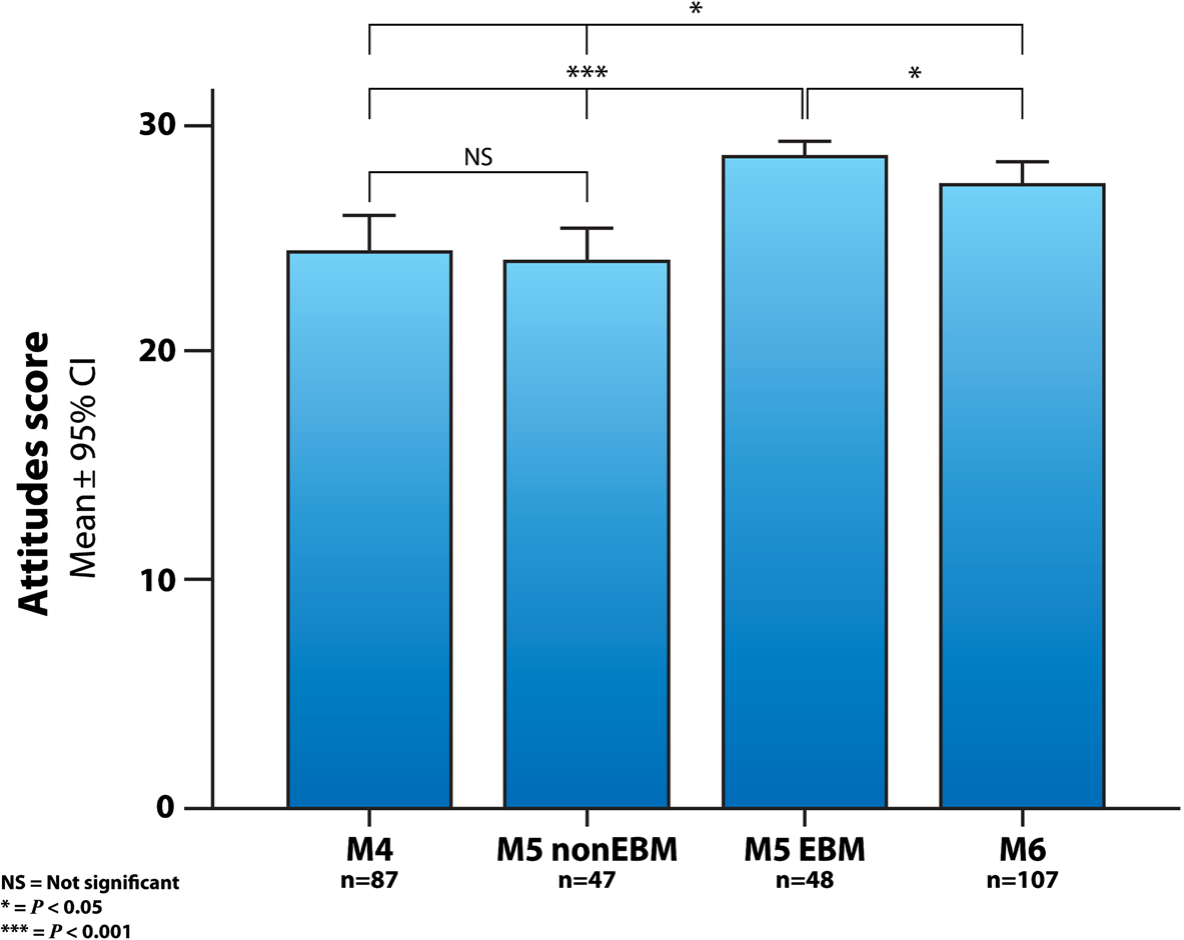
**Attitude scores.** Attitude scores in the different groups of medical students, measured with Taylor’s questionnaire. (M4=4^th^ year students with no evidence-based medicine training; M5 EBM and M5 non-EBM=5^th^ year medical students with and without the evidence-based medicine course; M6=6^th^ year students exposed to the evidence-based medicine course during the year prior to assessment; CI=confidence interval).

The EBM attitude scores measured with Taylor’s questionnaire are shown in Figure 5. The scores were similar between the groups that didn’t receive the EBM educational intervention, the M4 group had a score of 24.5±5.2 (mean±SD), and the M5 non-EBM group had 24.0±5.0 (*P*>0.05). The M5 EBM group had an attitude score of 28.7±2.2, higher than the M4 and M5 non-EBM groups (*P*<0.001). The M6 students had an attitude score of 26.7±3.6, higher than the control groups and lower than the M5 EBM group (*P*<0.05). Cohen’s *d* effect size for the comparison of M5 EBM vs. M5 non-EBM was 1.21 (Table 1).

**Table 1:** **Effect size (Cohen’s “*d*”) in critical appraisal confidence, attitude and knowledge scores when comparing the different medical student groups**

	**Critical appraisal confidence score Taylor instrument**	**Attitude score Taylor instrument**	**Knowledge score Taylor instrument**	**Knowledge score EBM MCQ test**
M5 EBM vs M4	1.05	1.06	0.91	4.06
M5 EBM vs M5 nonEBM	1.8	1.21	0.88	3.54
M6 vs M4	0.91	0.51	0.40	1.2
M6 vs M5 nonEBM	1.57	0.63	0.37	0.93
M6 vs M5 EBM	0.07	0.67	0.47	1.84

### Knowledge scores with Taylor’s instrument

**Figure 6:**
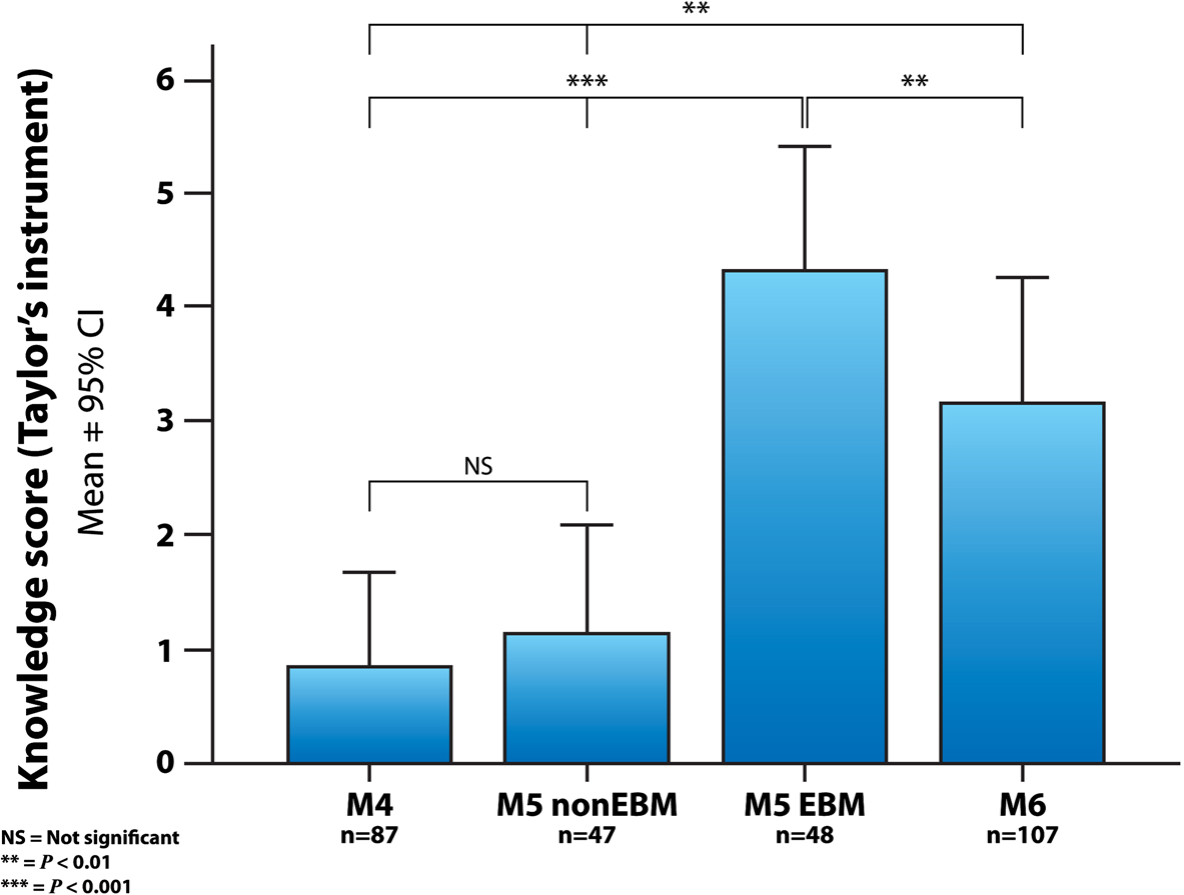
**Knowledge scores with Taylor’s instrument.** Knowledge scores in the different groups of medical students, measured with Taylor’s questionnaire. (M4=4^th^ year students with no evidence-based medicine training; M5 EBM and M5 non-EBM=5^th^ year medical students with and without the evidence-based medicine course; M6=6^th^ year students exposed to the evidence-based medicine course during the year prior to assessment; CI=confidence interval).

The results of the knowledge score measured with Taylor’s questionnaire are shown in Figure 6. The scores were similar between non-EBM groups, M4=1.06±3.16 (mean±SD), and M5 non-EBM=1.13±3.27 (*P*=0.91). The M5 EBM intervention group had a knowledge score of 4.21±3.73, higher than those of M4 and M5 non-EBM. The planned contrast in the main comparison between M5 EBM and M5 non-EBM showed that the intervention group had a higher knowledge score than the randomized control group (*P*<0.001). The M6 group had a knowledge score of 2.44±3.77, higher than both control groups, (*P*<0.01), but lower than M5 EBM (*P*<0.01). The effect size measured with Cohen’s *d* for the knowledge score main comparison of M5 EBM vs. M5 non-EBM was 0.88 (Table 1).

### Knowledge scores with EBM summative MCQ test

**Figure 7:**
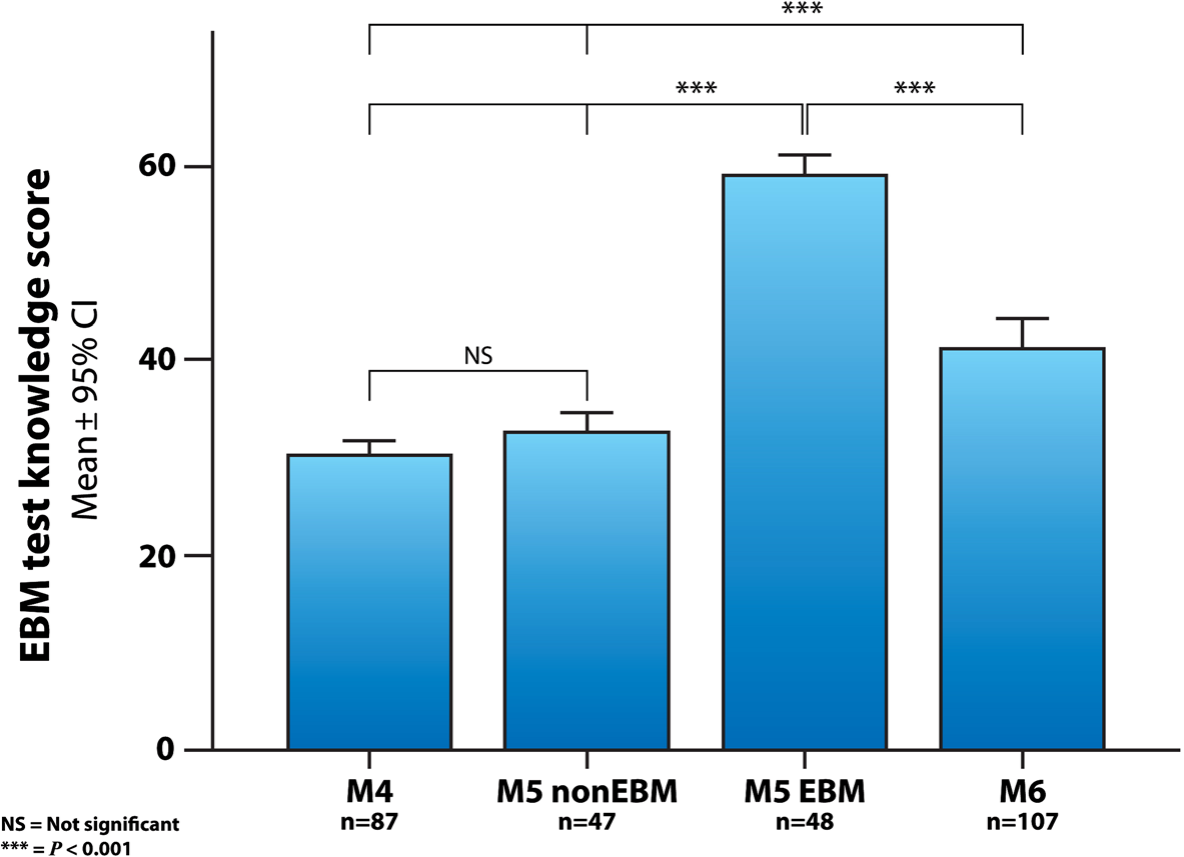
**Knowledge scores EBM test.** Knowledge scores in the different groups of medical students, measured with the 100 multiple-choice questions EBM test. (M4=4^th^ year students with no evidence-based medicine training; M5 EBM and M5 non-EBM=5^th^ year medical students with and without the evidence-based medicine course; M6=6^th^ year students exposed to the evidence-based medicine course during the year prior to assessment; CI=confidence interval).

The results of the 100-item MCQ EBM knowledge test are presented as percent-correct scores (Figure 7). The reliability of the test with Cronbach’s alpha was 0.72 in the M5 EBM group, and 0.83 in the M6 group. The scores were similar between non-EBM groups, M4=30.6±5.6 (mean±SD), and M5 non-EBM=32.6±6.6 (*P*=0.18). The M5 EBM group had a test score of 58.5±7.9, higher than M4 and M5 non-EBM. The planned contrast between M5 EBM and M5 non-EBM found that the educational intervention group had a higher knowledge score (*P*<0.001). M6 had a knowledge score of 41.0±10.9, higher than the control groups (*P*<0.001), but lower than M5 EBM (*P*<0.001). The effect size with Cohen’s *d* for the knowledge score main outcome comparison of M5 EBM vs. M5 non-EBM was 3.54 (Table 1).

## Discussion

This research study presents experimental evidence that an EBM educational intervention in medical students increases attitudes, knowledge and self-reported critical appraisal skills, in the setting of a developing country medical school.

The research design was a parallel-group randomized control trial, with a quasi-experimental static-groups comparison, to take advantage of a strong study design and its implications in terms of internal validity and the causal inferences that can be made of the results [24,25,33]. Recent studies and systematic reviews suggest that well-planned and educationally sound EBM interventions can have a reasonable impact on the abilities of the individuals that undergo these educational experiences [9,14,34].

There are not many published randomized controlled trials that study the impact of EBM education and very few from developing countries [9-12,14]. Some of the randomized trials did not find an effect of EBM educational interventions, which point to the need of continuing research in this area [35-37].

In the present study the educational intervention was one semester long, it was mandatory, and had a summative test, all these factors probably contribute to the magnitude of the findings in the randomized comparison. Almost all published studies have used only one assessment instrument, while our study used two evaluation tools, a published questionnaire with validity evidence designed to measure the effectiveness of evidence-based practice teaching, and an ad hoc objective test developed for the course summative assessment [29,30]. This characteristic of our study design provided an opportunity to concurrently validate an already published instrument and a new objective test developed specifically for our course, contributing to the body of literature supporting the validity of Taylor’s instrument.

We found an increase in critical appraisal skills, and in the positive attitude to evidence-based practice. These findings are similar to Ghali et al. [16], with a higher reported use of secondary journals and Cochrane Library systematic reviews. It is important to recognize that these are self-reports, the actual behaviour of the students in the use of these resources in their daily routines wasn’t directly measured.

In our study the answers to two questions related to the use of evidence (to keep up to date and to solve clinical problems) had a similar pattern of responses to our previous paper, as measured with Taylor’s questionnaire [21]. There was a higher reported use of the Cochrane Library and secondary journals in both items in the M5 intervention group, and a higher use of original research papers to solve a healthcare problem. It is apparent that all the students use frequently textbooks, Internet resources, teachers and residents as sources of information in health care, as previously reported [21]. These resources are readily available, and culturally accepted in the daily practice of medicine.

The use of the Cochrane Library and secondary journals was higher in our intervention group, which suggests that these resources were virtually unknown to the students before the course and that its reported use probably increased as a result of the educational intervention. Even though these EBM information resources have been extensively used in developed countries in the last decades, developing countries have been slower in adopting them as formal information elements, probably because of a lack of availability and misunderstanding of their potential use [38,39]. The Cochrane Library has been translated to Spanish by the Iberoamerican Cochrane Network, as the Cochrane Library Plus (http://cochrane.bvsalud.org), which should improve the availability and use of this resource in Spanish-speaking countries.

This study found that the EBM intervention improved the confidence of medical students regarding several aspects of critical appraisal skills, as well as statistical concepts relevant to the correct interpretation of published research findings. An interesting aspect of these results is that the medical students who weren’t exposed to the EBM course (M4 and M5 non-EBM), already had courses on Biostatistics and Scientific Methodology and nonetheless had lower scores in this outcome. Probably those courses didn’t have a substantial impact or it was short-lived. Other explanations could be that the previous courses on related subjects were given by non-clinicians and/or basic research scientists with no clinical orientation, having a minor effect on the EBM outcomes. The increase in critical appraisal skills is in agreement with several published reports of EBM in undergraduate students [15,16]. Other studies haven’t found a significant improvement in critical appraisal skills, probably due to several factors inherent to the complexity of educational research interventions in healthcare settings [35-37]. In our study the effect size immediately after the course in critical appraisal skills score was higher than 1.0, which can be interpreted as large using Cohen's classification [32]. A similar effect size was found when comparing the students that had the EBM course six months to one year before with the control group (Table 1).

It is important to recognize that self-perceived skills can overestimate true competence and performance, so these findings may not reflect the real critical appraisal and statistics skills of the medical students, although confidence in a skill is an important component of the performance spectrum [40,41].

The overall attitude score findings in our study are congruent with several published papers, showing an increase immediately after the course of about 17-20% [16,21,23,42]. The 6th year students attitude score was higher than the control group and the 4th year students, which suggests that the attitude change can still occur from six months to a year after the course. Our previous study found very similar attitude score values measured with the same instrument, which adds reproducibility evidence to the use of Taylor’s instrument for measurement of EBM attitude in our population of students [21]. It is noteworthy that some studies, including randomized controlled trials of EBM teaching, didn’t find a change in attitudes, probably due to the shorter duration of the workshops and related activities [36,37].

A major challenge of assessing EBM teaching is to demonstrate an increase in the “knowledge” of evidence-based clinical practice, since several disciplines intersect in the optimal use of scientific evidence (research methodology, biomedical informatics, biostatistics, clinical epidemiology) which integrate a large body of knowledge and facts. In this investigation, large effect sizes in the main randomized comparison (M5 vs. M5-nonEBM) were found in the EBM knowledge scores measured with Taylor’s questionnaire and the EBM MCQ test. The knowledge increase after the course was about 73% higher than the control group when measured with Taylor’s instrument, and 25.9% when measured with the EBM test. These increases can be interpreted as large when expressed as effect sizes using Cohen's classification, 0.88 and 3.54 respectively [32]. The fact that the changes were apparent when measured with two different instruments, adds validity evidence to the conclusion that the EBM course significantly improved the students’ knowledge base about EBM and its related concepts.

The EBM knowledge level was similar in the M4 and M5 non-EBM groups, which strongly suggests that the amount of EBM knowledge without a specific educational intervention is minimal even in the senior years of our medical school, and that there was no maturation threat to internal validity.

The significantly lower EBM knowledge scores in 6^th^ year students, in the time period of six months to a year after a similar intervention, suggests the possibility of knowledge decay, with decreasing amount of knowledge as time passes, unless continuous learning and practice occurs [43]. This difference in knowledge could be explained by the fact that our 6^th^ year measure was done in a different group of students, not the randomized 5^th^ year class, so it may not represent a true measure of knowledge decay but a difference in students' ability and it is uncertain how this would impact their use of EBM in clinical practice.

Other published randomized controlled trials of EBM educational intervention have produced conflicting results regarding knowledge change, with some of them showing minimal or no differences after the intervention [35-37] whereas others have found knowledge score increases of 36 to 58% [42,44]. These differences are probably due to the different nature of the educational interventions, their duration and the educational context (e.g. mandatory course). The use of effect size indices like Cohen’s *d* in EBM educational research publications could help visualize in a more standardized fashion the magnitude of the differences among studies, and promote reflection about the potential educational significance of the findings [45,46].

A limitation of the study is that it does not measure the actual competence and performance of EBM-related skills in a real clinical setting. Another potential limitation is related to the generalizability of the study, since the medical school has some particular characteristics because of its military nature, which could limit extrapolation to other medical schools. As with any implementation of a new course in a medical school, there was an intense interest from the course instructors to develop and implement as effective an educational intervention as possible, so there could be a tendency for confirmation bias. This can be expected in an education experimental study, where it is not possible to blind either the instructors or the students to the educational intervention. The data analysis was blinded in an attempt to decrease this bias. Another possible source of bias could be the Hawthorne effect, since students in the randomized intervention group were aware that they were being assessed on the course effectiveness, differently from the students that had the regular course previously [25].

## Conclusions

Our study has implications for the design, implementation and assessment of EBM educational interventions in developing countries. Firstly, it shows that EBM courses can be successfully implemented and embedded in a medical school’s curriculum. Secondly, it provides evidence that the course can improve knowledge, attitudes, critical appraisal confidence, and self-reported skills and behaviours about EBM and its related concepts, although the amount of knowledge that changes with time is still uncertain. And thirdly, it attests to the fact that using international test development standards can contribute to the development of a reliable instrument with evidence of construct validity for the measurement of EBM knowledge acquisition. The study findings contributed to the quality improvement process in the medical school, and provided data to be used in the planning and implementation of subsequent EBM courses. Educational planning will address its clinical links and vertical/horizontal integration with the rest of the curriculum (explicit and hidden), and more studies with rigorous follow-up should be undertaken to identify EBM competencies retention in the long-term. Published models and recommendations to increase the depth and duration of EBM learning should be taken into account when initiating educational interventions of this nature [47,48].

## Competing interests

The authors declare that they have no competing interests.

## Authors’ contributions

MS, LK and SM planned, designed and implemented the EBM course and the summative test, and applied the assessment instruments. MS, SD and AS participated in the design of the study and the statistical analysis. MS drafted the initial version of the manuscript. All authors read and approved the final manuscript.

## Pre-publication history

The pre-publication history for this paper can be accessed here:

http://www.biomedcentral.com/1472-6920/12/107/prepub
